# A cross-sectional needs assessment for a trauma-informed care curriculum for multidisciplinary healthcare providers

**DOI:** 10.1186/s12913-025-12568-1

**Published:** 2025-03-24

**Authors:** Dana C. Ross, Kaniz Fatema Farhat, Negar Sayrafizadeh, Annie K. Truuvert, Louloua Ashikhusein Waliji, Mahum Musheer, Julie Blair, Lesley Hughes, Sue MacRae, Simone N. Vigod, Sophie Soklaridis, Nancy McCallum

**Affiliations:** 1 Department of Psychiatry, Temerty Faculty of Medicine, University of Toronto, Toronto, ON Canada; 2 Women’s College Hospital, 76 Grenville Street, 7th floor, Toronto, ON M5S 1B2 Canada; 3 The Wilson Centre, University of Toronto, Toronto, ON Canada; 4 Centre for Addictions and Mental Health, 76 Queen Street, Toronto, ON Canada

**Keywords:** eHealth, Online, Virtual, Asynchronous, PTSD, Trauma, Healthcare education, Training, Mixed methods

## Abstract

**Background:**

Trauma-informed care (TIC) is a framework that recognizes the pervasive impact of trauma, aiming to enhance both patient outcomes and provider well-being. Given the high prevalence of trauma among individuals seeking healthcare, it is essential for healthcare providers (HCPs) to be trauma informed. However, standardized TIC curricula for training healthcare staff are lacking. This study assessed perceptions towards TIC among multidisciplinary HCPs, patients, and leadership staff at two urban hospitals in Canada.

**Methods:**

This mixed-methods prospective cross-sectional study employed Kern’s six-step approach for curriculum development. A needs assessment was conducted via an online questionnaire for HCPs and semi-structed interviews with individuals from the three participant groups: HCPs, patients, and leadership staff. The questionnaire assessed knowledge, skills, and attitudes regarding TIC. Semi-structured interviews explored perspectives on TIC, including curriculum priorities and potential implementation barriers. Findings informed the development of a virtual TIC curriculum, with iterative feedback collected to refine and assess its acceptability.

**Results:**

Among 106 HCP questionnaire respondents including Medical Doctors, Social Workers and Registered Nurses, 96 (90.6%) identified as women, and 97 (91.5%) as providers of direct patient care. Despite 93 (87.7%) having prior TIC education, 77 (72.6%) reported low confidence in applying TIC knowledge in clinical practice. Key perceived challenges to TIC training implementation included time constraints and lack of standardization across disciplines. A multimedia, self-paced course was the preferred solution. Thematic analysis of interviews with 28 participants (10 HCPs, 10 patients, 8 leadership staff) revealed six major themes: healthcare interactions, TIC implementation, training needs, system level barriers, curriculum preferences, and systems level improvements. Participants underscored the risk of re-traumatization to patients in healthcare settings without TIC and emphasized the need for universal TIC training for all staff.

**Conclusion:**

This study revealed a strong interest in a TIC course for multidisciplinary HCPs, supports the translation of knowledge into practice and incorporates a focus on cultural humility. Integrating insights from key stakeholders in this needs assessment phase resulted in the development of a TIC curriculum inclusive of diverse voices and viewpoints and strengthened the understanding of contextual factors that will support effective TIC implementation.

**Supplementary Information:**

The online version contains supplementary material available at 10.1186/s12913-025-12568-1.

## Introduction

The global prevalence of trauma has been well established, with experts identifying trauma as a major public health issue [3, 59, 60]. Individuals and communities who have experienced trauma in the form of physical, emotional, or sexual abuse, neglect, or witnessing abuse of others are more likely to suffer from adverse health outcomes [23, 66]. Furthermore, the effects of historical trauma, mass trauma, and systemic trauma are known to persist across generations, confirming that the consequences of trauma extend well beyond the time frame in which any singular event occurred [1, 11] Compared to the general population, in healthcare settings there is a higher prevalence of trauma among patient populations and healthcare providers (HCPs) [50, 90, 98]. Unfortunately, traditional healthcare delivery models are fraught with systemic barriers and inequities that can further exacerbate trauma responses and increase the use of resources, strain healthcare interactions, and contribute to burnout in HCPs [42, 54].

Trauma-informed care (TIC) has emerged as a framework that acknowledges the widespread prevalence of trauma in the general population [36, 38]. TIC promotes patient autonomy by using a harm reduction approach and offers sensitive practices for trauma screening, which minimizes triggers to prevent re-traumatization [12]. It also offers appropriate support and resources upon disclosure of trauma [5, 58]. The TIC framework offers healthcare staff an opportunity to implement responsive changes, resulting in more sensitive and compassionate approaches that lead to enhanced patient care outcomes such as reduced symptoms of post-traumatic stress disorder (PTSD), and improved overall functioning [17, 40, 95]. TIC is also an effective framework to support provider well-being by addressing burnout and secondary vicarious traumatic stress [28]. As a result, its use is associated with greater staff retention and job satisfaction [67, 78, 97].

Recent reviews highlight a growing body of literature on TIC education frameworks, yet the quality, content, duration, and delivery methods of existing curricula vary widely [45, 61, 83]. Often, TIC curricula are tailored to specific healthcare providers or settings, such as medical residents, primary care providers, nurses, or emergency room staff [14, 39]. However, there is increasing recognition of the need for TIC education that is inclusive of multidisciplinary teams, reflecting the collaborative nature of healthcare and the benefits of organization-wide training [15, 56, 79, 85]. Studies on TIC education for interprofessional providers have demonstrated positive outcomes, particularly in attitudes and behaviours related to TIC [18, 24, 65, 73]. Despite this identified need, barriers like time constraints, access, cost, and the challenges of training multidisciplinary teams persist [6, 62, 87]. There is also a growing call for increased patient engagement and collaboration in the curriculum development process to ensure diverse experiences and perspectives are incorporated [4, 44].

Existing TIC training programs are often delivered through synchronous or hybrid formats that typically include traditional in-person workshops or live virtual sessions [64, 83]. While these programs have proven effective in healthcare settings, showing high rates of satisfaction and improving knowledge and confidence, they tend to be resource-intensive and difficult to scale [17, 32, 68]. Following the COVID-19 pandemic, fully asynchronous or primarily self-directed virtual courses have gained traction, offering busy professionals the flexibility to complete training at their convenience [25, 43, 71]. Emerging evidence suggests that asynchronous training formats can significantly boost TIC-related knowledge and confidence, though further research is needed to identify optimal strategies for curriculum development [8, 46].

Using a mixed methods approach, this study assessed the needs and preferences for a curriculum on TIC for HCPs at two urban academic hospitals. The primary objective of the study was to conduct a needs assessment to evaluate key stakeholders’ (i.e., HCPs, patients and leadership staff) perceptions about the clinical and educational needs for a curriculum on TIC developed for multidisciplinary HCPs. The primary outcome measures included the evaluation of healthcare providers’ TIC-related knowledge, skills, and attitudes, as well as their preferences for a TIC course. The secondary objective was to use the quantitative questionnaire data from HCPs, and qualitative data from individual interviews with HCPs, patients and key stakeholders, to map out the content for a TIC curriculum outline, including learning outcomes and objectives. The initial curriculum outline, developed from the needs assessment, was evaluated for acceptability by gathering feedback from interview participants.

The anticipated future users of the course are multidisciplinary HCPs who are seeking specific training to integrate TIC principles and practices into their workplace. The findings of this study will expand knowledge on TIC curriculum needs and inform the future development and evaluation of a TIC course.

## Methods

### Conceptual framework

We used Kern’s six-step method for curriculum development [92] to guide the process for this study. The goal was to create a curriculum outline tailored to the needs and learning gaps of HCPs and shaped by data from patients and staff in hospital leadership roles. Kern’s six-step model for curriculum development starts with identifying the problem and conducting a general needs assessment to elucidate the gap between the existing and ideal approaches to addressing the stated problem that will be addressed by the education intervention (*Kern’s step 1: Problem Identification and General Needs Assessment*) [16]. The problem identified was the lack of accessible, scalable and affordable TIC training for multidisciplinary HCPs that could be implemented widely across a healthcare organization. A general needs assessment was conducted through informal conversations with HCP colleagues and learners and reviews of past literature. The focus of the current study was on Kern’s step 2 (*Targeted Needs Assessment*) and Kern’s step 3 (*Development of Goals and Learning Objectives).* In a future phase of the study, we will explore Kern’s step 4 involving incorporation of specific educational strategies in alignment with the objectives to attain the desired outcomes and step 5 which corresponds to implementation of the intervention aided by applicable personnel and resources [16]. The last step of the six-step model evaluates the success of the curriculum by generating feedback from individual participants [16]. While the full evaluation of the curriculum’s implementation will be assessed in a future phase, in the current study, we conducted a formative evaluation by circulating the TIC curriculum outline among individual interview participants to generate feedback and evaluate acceptability and to make iterative changes to finalize the outline.

### Study design and participants

A mixed-methods, prospective design was employed, consisting of a needs assessment questionnaire for HCPs and semi-structured individual interviews with HCPs, patients, and leadership staff at two academic hospitals located in Toronto, Ontario: Canada’s largest urban centre. The study sites included Women's College Hospital (WCH), which is an ambulatory teaching hospital and The Centre for Addiction and Mental Health (CAMH), which is the largest mental health teaching hospital in Canada. The study initially included nurses, social workers (SWs), and psychiatrists in the HCP group, but later expanded to all healthcare providers in the two hospitals to incorporate a broader range of perspectives. The leadership staff at WCH and CAMH included managers, and leaders in healthcare and education. Patient participants were recruited from the Trauma Therapy Program (TTP) at WCH and they all self-reported experiencing childhood interpersonal trauma before the age of 18, making them well-positioned to provide insights into their experiences with TIC across the healthcare system. All study participants were 18 years of age or older and had proficiency in reading and understanding English. Purposive sampling was used to select HCP participants with experience in patient-facing, clinical care for both the qualitative and quantitative components [89]. As such, feedback was generated from HCPs with richness of experience, knowledge of or training in TIC, or exposure to healthcare environments where TIC would be applicable [74].

### Kern’s step 2: targeted needs assessment

A targeted needs assessment identifies both the needs, preferences, and available resources of the targeted learner population and of the stakeholders [16]. The targeted needs assessment included an online anonymous needs assessment questionnaire distributed among HCPs to assess knowledge, skills, and attitudes about TIC. The questionnaire was followed by individual interviews with HCPs, patients, and leadership staff.

#### Needs assessment questionnaire

Guided by the study’s aims and using relevant literature, the research team developed the questionnaire. It consisted of multiple-choice questions, 5-point Likert scale responses, and demographic questions. We pilot-tested the questionnaire via email with a small group of HCPs in the TTP at WCH. After integrating their feedback, the questionnaire was finalized, approved by the Research Ethics Board, and distributed. The inclusion criteria for questionnaire respondents required participants to be employed at WCH or the CAMH.

The questionnaire was distributed to HCPs in 18 departments (see Table 1) across WCH and CAMH between September 2022 and May 2023, including nursing, social work, psychiatry, family practice, and medical and surgical specialty departments. The recruitment email comprised a brief study description, definition of TIC, a description of the questionnaire content and estimated time of completion. REDCap (Research Electronic Data Capture), a secure, web-based application designed exclusively to support data capture for research studies, was used to provide a link in the email to the questionnaire with an embedded consent form [94]. The link to the needs assessment was also distributed to staff and learner groups at meetings. Potential participants were directed to the consent form upon clicking on the REDCap link and were able to complete the questionnaire after providing consent. Participants could enter their contact information after completing the questionnaire for a chance to win one of three $25 gift cards.

**Table 1:** Demographic data obtained from anonymous questionnaire distributed among HCPs

Demographics Characteristics	Total *N* = 106 *n* (%)
Age group (in years)	
18–24 years old	2 (1.9)
25–34 years old	32 (30.2)
35–44 years old	40 (37.7)
45–54 years old	17 (16)
55–64 years old	15 (14.2)
65 years or older	0 (0)
Gender	
Woman	96 (90.6)
Man	5 (4.7)
Non-binary	5 (4.7)
Two-Spirit, Transgender, Prefer not to say, Self-identify, None of these apply to me	0 (0)
Race/Ethnicity^a^	
White North American	43 (40.6)
White European	17 (16)
South Asian	11 (10.4)
Jewish (i.e. Ashkenazi, Sephardi, Maghrebi, etc.)	10 (9.4)
East Asian	8 (7.5)
Middle Eastern or Arab	6 (5.7)
Prefer not to say	5 (4.7)
Latin American or Hispanic	5 (4.7)
Southeast Asian	4 (3.7)
Other	3 (2.8)
Black (Caribbean, North American, African)	3 (2.8)
Indigenous (First Nations, Inuit, Métis)	0 (0)
Don't know	0 (0)
Missing data	1 (0.9)
Site of practice^b^	
Women’s College Hospital	87 (82.1)
Centre for Addiction and Mental Health	20 (18.9)
Missing data	1 (0.9)
Role	
Staff	88 (83)
Student	16 (15.1)
Other	2 (1.9)
Employment status^c^	
Full-time	75 (70.8)
Student/learner	14 (13.2)
Part-time	11 (10.4)
Casual or time-limited contract	6 (5.7)
Prefer not to say	2 (1.9)
Other	0 (0)
Missing data	1 (0.9)
Profession	
Other^d^	40 (37.7)
Nursing	30 (28.3)
Social Work	26 (24.5)
Psychiatry	10 (9.4)
No. of years in current profession	
Less than 1 year	4 (3.8)
1–5 years	25 (23.6)
6–12 years	32 (30.2)
13–20 years	26 (24.5)
Over 20 years	19 (17.9)
Direct patient care involvement	
Yes	97 (91.5)
No	8 (7.5)
Other	1 (0.9)

^a^Column values (n) do not add up to 106 as HCPs selected more than 1 Race/Ethnicity

^b^Column values (n) do not add up to 106 as HCPs employed at both WCH and CAMH

^c^Column values (n) do not add up to 106 as HCPs selected more than 1 Employment Status

^d^Other departments/clinics where the questionnaires were distributed included: Cardiology, Centre for Headache, Dermatology, Endocrinology, Gastroenterology, Respirology, Perioperative Services, Gynecology, Medicine, Family Medicine, General Internal Medicine as well as the Medical Affairs, Family Practice Health Centre, Substance Use Services, and After Cancer Treatment Transition Clinic

To facilitate recruitment, we requested that administrative personnel provide the number of HCPs on their listservs.

#### Individual interviews

Semi-structured, virtual, 30 to 45-min one-on-one interviews with patients, HCPs, and leadership staff were conducted by research assistants. The interview guides consisted of open- and closed-ended questions developed by the research team using previous literature on TIC education (see Additional file 1–3). Recruitment for potential individual interview participants began September 2022 and continued until October 2023. Patient participants for the individual interviews were recruited via flyer handouts posted in therapy groups in the TTP at WCH. Those who wished to participate contacted the study research staff who then explained the study, reviewed eligibility criteria, and conducted informed consent procedures. HCP participants were invited to enter their contact information at the end of the needs assessment questionnaire if they were interested in participating in the individual interviews. The research staff contacted those who signed up, reviewed the interview process, and conducted informed consent procedures prior to conducting the virtual interview. Leadership staff were identified by the research team and recruited via email by a research assistant. If leadership staff indicated interest, informed consent procedures were conducted prior to the virtual interview. All individual interview participants received a $30 digital gift card. Past research on general qualitative analysis of interview-based data indicates that data saturation is attained when no new information or themes emerge with additional interviews [82]. Smaller sample sizes in qualitative interviews provide richer data given the in-depth nature of individual interviews. Qualitive analysis of interview-based data collection indicates a sample size of 9–17 to be adequate to research data saturation [41]. Using the concept of information power in qualitative research, we anticipated conducting a total of approximately thirty individual interviews with HCPs, patients, and hospital leadership staff [91].

### Data analysis

Responses were linked to unique study identifiers on REDCap, enabling the data to be downloaded in a de-identified format for analysis. A descriptive analysis was conducted on slider-based responses reflecting HCPs' interest in ten TIC topics. The data was processed to calculate the mean value across the dataset. De-identified interview recordings from HCPs, patients and leadership staff were organized by unique study identifiers and transcribed by a professional transcription service provider. An explanatory sequential design was employed by using the qualitative data from individual interviews to explain and expand on data that was gathered through the quantitative questionnaire [30]. De-identified interview data was analyzed utilizing a data-driven inductive thematic approach [22]. Three research team members analyzed the transcripts independently. The analysis process began with familiarizing with the data by reading each transcript line by line, followed by highlighting concepts and associating meanings to the text to generate a list of codes. The codes and data extracts were collated, and potential themes were organized into overarching themes. Final themes were established through a collaborative review by the three research team members who compared all the themes generated in their individual analysis and then collected relevant themes to match with data extracts, to determine overarching themes. This process ensured that the analysis was accurate, consistent and reliable [81].

### Kern’s step 3: goals and learning objectives

#### Curriculum outline

In the third step of the model, goals and objectives were established based on the needs assessment conducted in the previous stages to help define the educational and evaluation strategies for the intervention [16]. The study team planned to create an initial draft of a curriculum outline for a TIC course based on the results from the needs assessment and then sought feedback by circulating the initial draft via email among interview participants who consented to provide feedback.

#### Ethics

This initiative was formally reviewed and approved by the Women’s College Hospital (WCH) Research Ethics Board (#2022–0032-E).

## Results

### Needs assessment survey

A total of approximately 1159 HCPs across the 15 departments received the questionnaire via departmental listservs. There were 120 HCPs who consented to participate in the quantitative survey, 106 (88.3%) of whom completed the questionnaire (Table 1). The median age range of the respondents was 35–44 years with the majority being women. Given that most HCPs had spent a significant number of years working in their current profession (median = 6–12 years) and were full-time staff members, the majority had the opportunity to gain experience with direct patient care. Respondents represented a diverse group of professionals including but not limited to nurses, SWs, Medical Doctors (MDs), occupational therapists, Registered Psychotherapists, family physicians, and pulmonary function technologists.

### Current level of TIC knowledge and need

Based on data generated from 106 HCPs completing the needs assessment questionnaire a gap in TIC training was highlighted (Table 2). Of these 106 HCPs, 12.3% (*n* = 13) reported no prior TIC training, 39.6% (*n* = 42) received 1–5 h of training previously, 18.9% (*n* = 20) 6–10 h; 6.6.% (*n* = 7) 11–20 h and 22.6% (*n* = 24) received more than 21 h. Almost all respondents (*n* = 102) identified TIC to be very or somewhat important to their role and 91.5% of the HCPs indicated interest in taking a virtual TIC course. While 27.4% responded that they are very confident about their current level of understanding of what is meant by the term ‘TIC’, and 23.5% feel very comfortable interacting with clients who have a history of trauma, only 14.2% felt very satisfied with their current level of training on TIC.

**Table 2:** Likert scale questions regarding attitudes and confidence about TIC

Questions HCP ratings, n (%)	Very important/ interested/ confident/ comfortable/ satisfied	Somewhat important/ interested/confident/ comfortable/ satisfied	Neutral	Somewhat unimportant/ uninterested/not confident/ uncomfortable/ dissatisfied	Very Unimportant/ uninterested/not confident/ uncomfortable/ dissatisfied
Importance of TIC to your role or area of practice	86 (81.1)	16 (15.1)	4 (3.8)	0 (0)	0 (0)
Level of interest in taking a virtual course on TIC	72 (67.9)	25 (23.6)	6 (5.7)	2 (1.9)	1 (0.9)
Confidence about your current level of understanding of the term TIC	29 (27.4)	42 (39.6)	21 (19.8)	13 (12.3)	1 (0.9)
Comfort interacting with clients who have a history of trauma	25 (23.5)	57 (53.8)	14 (13.2)	9 (8.5)	1 (0.9)
Satisfaction with your current level of training on TIC	15 (14.2)	40 (37.7)	22 (20.8)	21 (19.8)	8 (7.5)

When asked whether their work environment incorporates TIC principles and/or practices, 41.5% (*n* = 44) indicated ‘yes’; 47.2% (*n* = 50) ‘somewhat’; 8.5% (*n* = 9) unsure’, and 2.8% (*n* = 3) selected ‘no’. Of the 106 respondents, 56.6% (*n* = 60) believed they themselves incorporate TIC principles and/or practices in their work, 35.8% (*n* = 38) indicated ‘somewhat’; 6.6% (*n* = 7) were ‘unsure’, and 0.9% (*n* = 1) mentioned no incorporation of TIC in their work.

### Topics of interest

HCPs were also asked to rate their interest in ten TIC topics. A list of topics was provided, and respondents used a slider to quantify their interest on a scale from 1 to 100, corresponding to ‘Not Interested,’ ‘Somewhat Interested,’ and ‘Very Interested.’ Results are outlined in Table 3 below. Furthermore, respondents were also asked to suggest any topic that was not included in the list and that they would like to see in a virtual trauma-informed care course. The suggestions emphasized the need for trauma-informed education, covering the impact of trauma on chronic diseases, substance use, poverty, PTSD vs. complex trauma, and practical strategies for trauma recovery.

**Table 3:** TIC topics of interest identified and ranked by HCPs completing the questionnaire

Rank Order	TIC Topics	Mean
1	How to ask about trauma, respond to patient disclosures, and reduce the potential for retraumatizing when delivering services	86.5
2	How to incorporate principles of equity, diversity, inclusion, and belonging into trauma-informed care practices, including performing culturally sensitive assessments	84.9
3	Trauma-informed care skills and strategies for general use in clinical work (e.g., grounding tools to manage emotional dysregulation and flashbacks)	84.1
4	How trauma diagnoses and symptoms can overlap with other mental health diagnoses	82.1
5	Understanding that common mental health symptoms can be understood as trauma coping strategies	80.5
6	Overview of trauma-informed care treatment modalities	79.3
7	How to implement trauma-informed care in the workplace on a systemic or organizational level	79.3
8	How to correctly use instruments intended to screen and assess for trauma and trauma-related symptoms	77. 7
9	Neurobiology of trauma-related symptoms and responses	77.4
10	Knowledge of trauma: definition, prevalence, impact, and recognition	69.3

### Course preferences

When asked how much experience HCPs have with taking virtual courses that are asynchronous, 52.8% (*n* = 56) indicated ‘lots of experience’, 41.5% (*n* = 44) ‘some experience’, 4.7% (*n* = 5) ‘little experience’ and 0.9% (*n* = 1) mentioned ‘none’. Almost all (*n* = 101, 95.3%) indicated that they would engage in a trauma-informed care course if it was delivered virtually (as opposed to in-person), and a majority (*n* = 88, 83.0%) indicated that they would engage in a trauma-informed care course if it was delivered asynchronously (i.e., reviewing educational material on your own time), as opposed to synchronously (i.e., lecture-style group learning). When asked how much time in total would be acceptable for HCPs to dedicate to completing a virtual course on trauma-informed care, 18.9% (*n* = 20) indicated ‘1 h’, 41.5% (*n* = 44) ‘2–3 h’, 31.1% (*n* = 33) ‘4–6 h’ and 8.5% (*n* = 9) chose ‘7–10 h’.

### Individual interviews

#### Recruitment, retention, and demographics

Fifty potential participants were contacted for the individual interviews. Twenty-three HCP participants indicated interest in receiving more information about the individual interviews by checking a box on the questionnaire. Seventeen were contacted and participated, at which point data saturation was reached, and no further interviews were conducted. Approximately 80 patients received the recruitment flyer, and 13 patients signed up via a recruitment flyer requesting to be contacted with more information about the study and all were contacted by the research team. Twenty leadership staff were emailed directly about the study and 9 expressed an interest in participating.

A total of 29 participants consented and were enrolled (11 HCPs, 10 patients, 8 leadership staff) and 28 participants completed the individual interviews (10 HCPs, 10 patients, 8 leadership staff). The retention rate for individual interviews was 96.6%. One person provided consent but did not complete the interview. Data saturation was obtained with 28 interviews.

#### Qualitative results

Out of the 28 interview recordings, one patient and one leadership staff interview recording were not transcribed due to the corrupted and irretrievable audio files. As such, 26 interview recordings (10 HCPs, 9 patients, 7 leadership staff) were successfully de-identified, transcribed and thematically analyzed (Table 4).

**Table 4:** Demographic data obtained from individual interviews with HCPs, patients, and leadership staff

Demographic Characteristics	Total *N* = 26 n (%)
Age range (in years)	
18–34 years old	5 (19.2)
35–54 years old	14 (53.8)
55–74 years old	5 (19.2)
Other	1 (3.8)
Missing data	1 (3.8)
Gender	
Woman	23 (88.5)
Non-binary, Self-identify, Gender queer, Two-Spirit, Other	2 (7.6)
Prefer not to say	1 (3.8)
Man	0 (0)
Missing data	1 (3.8)
Ethnic/Racial Group	
White or European	20 (76.9)
Black or Afro-Caribbean	2 (7.7)
Jewish	2 (7.7)
South Asian or Indian	2 (7.7)
Other _Biracial	2 (7.7)
Middle Eastern or Arab	1 (3.8)
Latino or Hispanic	1 (3.8)
East Asian or Asian	0 (0)
Indigenous (First Nations, Inuit, Métis)	0 (0)
Prefer not to say	0 (0)
Don’t know	0 (0)

### Healthcare providers

Two main themes identified from the HCP transcript analysis are 1) *‘Training Needs’* and 2) *‘System Level Barriers’* (Table 5).

**Table 5:** Thematic analysis of healthcare provider interviews

Themes	Subthemes
1. Training Needs	1. Content and strategies
	2. Course engagement
2. System Level Barriers	1. Time constraints
	2. Gap in TIC training
	3. Adaptable policies and procedures

#### Theme 1. Training needs

HCP needs and preferences from TIC training are captured under the first overarching theme ‘*Training Needs*’ with the subthemes being ‘*Content and strategies’* and *‘Course engagement’.*

In the subtheme ‘*Content and strategies’,* providers indicated their lack of understanding of what trauma means and expressed willingness to understand how the meaning of trauma has evolved to encapsulate various adverse experiences.

*“I just think that type of training that’s like super sense-making of people’s trauma, de-pathologizing, very compassionate, is so needed in our healthcare system.” [HCP 018].*

HCPs understand the importance of approaching all care with a TIC lens but expressed the need to learn how and when to ask about trauma and respond to disclosures in a sensitive manner.

*“I think a lot of us feel we don’t have the expertise to really manage someone who may be ready to talk about it or has been recently traumatized and we don’t know what to do with them afterwards.” [HCP 003].*

HCPs feared that their lack of skills to manage trauma-related symptoms might accidentally harm patients. They identified this as a barrier to addressing trauma during client interactions. Specifically, respondents expressed the willingness to learn practical TIC skills and apply them in their practice.

*“I think that what is lacking are the actual skills to provide treatment intervention for trauma, so kind of moving beyond just the knowledge of trauma-informed care” [HCP 010].*

The second subtheme, ‘*Course engagement’* captures HCPs’ preferences for an asynchronous course on TIC. While providers reached consensus on the feasibility of a self-paced course that can be accessed at their own convenient time, they also expressed the need to have a course that is interactive as well as one that provides an opportunity for content clarification. Respondents identified their preference to have various forms of multimedia like animation, simulations, podcasts and videos incorporated in a course to engage learners. The importance of having reflective exercises was highlighted to help learners grasp the application of knowledge.

*“Animation, something that is speaking and also words pop up, that way it’s visually appealing and informative and it’s not just me reading slides.” [HCP 020].*

#### Theme 2. System level barriers

The second major theme ‘*System Level Barriers*’ captures the barriers HCPs identified that hinder the delivery of trauma-informed care, along with recommendations to support TIC implementation. The subthemes include ‘*Time constraints’*, ‘*Gap in TIC training’*, and ‘*Adaptable policies and procedures’*. 

The majority of the respondents identified time constraints as a significant barrier to learning about TIC. As such, an asynchronous course with a flexible deadline is deemed feasible to fit into their busy schedule. Such a delivery format will also allow greater comfort and enable learners to focus better.

*“Yeah, overall, I think it’s a great tool, and having an asynchronous course can work very well for many health care professionals, especially with the constraints from time and so on.” [HCP 022].*

*“Well, the strength can certainly be that you could finish this on your own time when you’re not trying to squeeze it in between different things. You actually can pay attention and set time aside to actually do the learning well. It could more convenient too if you can just do it virtually…What I mentioned before about maybe just feeling more comfortable or safer to do it virtually, so you’re not embarrassed or you’re not feeling uncomfortable with other group participants or something like that.” [HCP 003].*

Another barrier identified was ‘*Gap in TIC training’*. Most respondents mentioned not receiving a formal education on TIC, rather their learning is based on professional development workshops and individual research.

*“I think this is really important and I think it’s something that obviously we should be doing in healthcare. Mental healthcare especially, it should be trauma informed.” [HCP 018]*.

Although providers understand the concept of TIC, they lack practical application skills and thus lack the confidence to practice TIC.

*“What am I worried about it? Not knowing what to do, not knowing how to respond. Accidentally harming them. Yeah, I think those are the big ones. Not being skillful and being too rushed. And not having anybody to conceptualize things with.” [HCP 008].*

Lastly, the subtheme ‘*Adaptable policies and procedures’* was generated as a recommended approach to address institutional barriers highlighted by HCPs. Notably, lack of identified resources and training prevents HCPs from learning about TIC. Further difficulties arise in identifying appropriate trauma resources to which to refer patients.

*“Trying to access mental health resources is like banging your head against a brick wall these days” [HCP 003].*

Last but not the least, respondents identified time-limited patient visits as a barrier to care for trauma-affected clients. Not only are providers unable to give as much time needed to a client, but they are also unable to see them as frequently as needed.

*“Time limitations. The service here is time limited. So, I can be a bit rushed, and that doesn’t really meet the person where they’re at.” [HCP 008].*

### Patients

Two overarching themes emerged in the patient interviews: *1) Healthcare Interactions (with providers) and 2) TIC Implementation* (Table 6).

**Table 6:** Thematic analysis of patient interviews

Themes	Subthemes
1. Healthcare interactions	1. Trauma-informed language and approach
	2. Enhanced provider knowledge about gender-based violence and female health
	3. Building rapport and tailoring care
2. TIC implementation	1. Lack of trauma education
	2. Standard procedures when providing TIC

#### Theme 1. Healthcare interactions

The first overarching theme ‘Healthcare Interactions’ highlights patients’ expectations from providers learning about and delivering TIC, encompassing three subthemes: ‘Trauma-informed language and approach ‘, ‘Enhanced provider knowledge about gender-based violence and female health’ and ‘Building rapport and tailoring care’.

The first subtheme *‘Trauma-informed language and approach’* highlights patients’ expectations about receiving trauma-informed care across diverse experiences with healthcare providers. Patients underlined the need for their providers to incorporate sensitivity, empathy and mindful language into their practice. Providers should be aware of the power differential in a provider–client relationship and approach care from a place of openness and non-judgment.

*“There’s a power differential right out of the gate, because one is asking for help and already that is a very tough place to be as a trauma survivor, to ask for help for anything.” [Pt 015].*

Patients further shared concerns about being dismissed by providers when presented with trauma-related symptoms and expressed the need for providers to have better understanding of how trauma might present across people from different cultures and backgrounds when conducting trauma screenings.

*“Just teach them how to be non-threatening… that’s important how you approach it [asking about trauma] to make someone feel safe.” [Pt 021].*

The second subtheme ‘*Enhanced provider knowledge about gender-based violence and female health*’ represents patients’ need for providers to understand how trauma impacts female health specifically and to approach care with specific understanding, sensitivity and openness.

*“I think there is a bigger impact [on women], and it [trauma] is impacting women because our healthcare does not support women as a whole” [Pt 012].*

Patients expressed concerns about not receiving TIC especially from male healthcare providers and emphasized the need to equip them with appropriate knowledge on gender-based violence and the unique features of trauma in women to avoid re-traumatization.

*“He didn’t listen, and I had a full-on flashback…I thought the doctor was trying to hurt me, I was really scared and that was really re-traumatizing” [Pt 002].*

The third subtheme ‘*Building rapport and tailoring care’* is another major aspect that was underlined by patient respondents. Patients highlighted the need to have choice and collaboration in their treatment.

*“Again, just making it safe, knowing what kind of context your patients are coming from, to me that seems like a moral imperative.” [Pt 015].*

Patients reported having autonomy in their treatment would enable them to feel safe and comfortable. A lack of control and autonomy undermines the sense of safety among patients.

*“If you’re not giving someone any options and you’re telling me that this is the only option you have and I’m just going to fix you in a way that already terrifies someone like me…it was almost like a non-option, and it was like you don’t really care to understand where I’m coming from” [Pt 021].*

*“People forget those kinds of things or don’t think it’s important asking somebody before you touch them, telling people exactly what’s going to happen and kind of like letting people… have autonomy over their bodies” [Pt 002].*

#### Theme 2. TIC implementation

The second major theme, *TIC Implementation’* represents perceived barriers to the implementation of TIC and patient expectations of TIC. The subthemes include *‘Lack of trauma education’* and ‘*Standard procedures when providing TIC.’*

The first subtheme, *‘Lack of trauma education’* underscores the importance of providing widespread trauma knowledge to healthcare providers. Not knowing how trauma impacts patients puts them at risk of misdiagnosis and can hinder access to appropriate care. Patients expressed the need for providers to have knowledge about trauma symptoms and how certain behaviors can be coping mechanisms for trauma survivors.

*“I guess just in general learning more about the way that symptoms would present differently in someone who has been through trauma, especially I think sort of learning to look for signs that someone is either uncomfortable or something when they’re not going to necessarily show it.” [Pt 025].*

Providing TIC education to all healthcare staff is a system level need that highlights the important role that healthcare organizations and institutions play in equipping all staff with the knowledge and understanding needed to engage in TIC.

*“We need front desk staff in those offices to be much more aware of workarounds, and they’re not. It’s one thing for a doctor to provide trauma-informed care but I also think that the office managers need to be looped into that as well, people who are taking calls on the phone. Because it has not been my experience.” [Pt 015].*

The subtheme ‘*Standard procedures when providing TIC’* indicates patients’ expectation from healthcare settings to have TIC policies in place. Patients wanted providers to have a standard procedure when charting their trauma history to maintain confidentiality and to practice sensitivity when sharing their personal history with other providers. They added that they would feel comfortable with their providers knowing that they have a trauma history, with the option of not having to share the details, if they are not comfortable doing so.

*“I think as long as if it was like a more general question and not going into a lot of detail or specifics, I think I would actually be happy with that because I think that would allow me to be comfortable knowing that they are aware that I have traumatic past experiences.” [Pt 026].*

Patients expressed that providers do not need to have details of trauma narrative to provide TIC. Lastly, respondents indicated the need to have accountability for providers who fail to practice TIC.

*“Having some form of accountability is something that I think is sorely lacking.” [Pt 025].*

### Leadership staff

The two main themes generated from leadership staff (LS) transcript analysis are *1) Curriculum preferences* and *2) Systems Level Improvements* (Table 7).

**Table 7:** Thematic analysis of leadership staff interviews

Themes	Subthemes
1. Curriculum preferences	1. An accessible and interactive curriculum with an attainable time commitment
	2. A customizable curriculum applicable to healthcare staff at all levels in an organization
2. Systems Level Improvements	1. TIC training for all staff
	2. Socially conscious
	3. Organizational barriers

#### Theme 1. Curriculum preferences

The first subtheme that emerged is ‘*An accessible and interactive curriculum with an attainable time commitment*.’ Leadership staff recommended the creation of an asynchronous course which is short and concise. They emphasized incorporating aspects to generate course engagement and facilitate learning.

*“Something that people can access when they have time, so something that can be consumed in short pieces. And what I mean by that is they might have, at the most, an hour to be able to engage in that kind of learning, and I would say just under an hour.” [LS 034].*

*“Have some kind of discussion board or some type of opportunity to share their reflections or concerns or questions. Because otherwise they’re left with all this thinking and nothing to do with it.” [LS 034].*

Further recommendations for the TIC training are captured under the second subtheme ‘*A customizable curriculum applicable to healthcare staff at all levels in an organization’*. Leadership staff mention that highlighting the importance of the training and its impact on learners’ day-to-day practice will motivate them to complete the course.

*“Motivation, for people to understand the importance of how this is going to benefit their work…So, being very clear on why it’s important and why this is going to help them provide better care and improve their satisfaction as well.” [LS 045].*

In terms of taking the course themselves, leadership staff wanted to learn about resources to which to refer to patients. Furthermore, additional resources should be provided if learners seek more information on a specific topic. Lastly, respondents indicated that specific versions of the TIC training should be created that cater to specific healthcare staff roles and needs.

*“Yeah, maybe there’s an option for the highlights version and then the in-depth version, for individuals who are, you know, providing hands-on care for people.” [LS 035].*

#### Theme 2. Systems level improvements

The expected outcome of having universal TIC training is captured under the subtheme ‘*TIC training for all staff*’. Leadership staff expect that having all staff members trained in TIC will facilitate improved patient outcomes as well as staff well-being.

*“I think TIC training should be accessible to all levels.” [LS 032].*

Further emphasis was put on enabling educational opportunities for not only staff but also student learners in the organization. Respondents indicated that equipping providers with appropriate knowledge and resources will enable them to be confident in practicing TIC and thus allow them to feel satisfied in their role.

*“Anything that helps you understand your patients better is going to improve your ability to connect with the patient and probably improve the patient outcome. There’s a benefit for both." [LS 045].*

Another subtheme identified from the analysis was ‘*Socially conscious’* which underlines the importance of building training with a focus on equity, diversity and inclusion. They highlighted the need to incorporate information on how trauma intersects with marginalization and cultural differences.

*“There needs to be a certain amount of generic information, but I think some of it has to be very specifically targeted towards populations at risk.” [LS 037].*

*“Have TIC training intersected with being anti-racist, how it interfaces with equality and oppression. We’re all trying to understand collapses in the system, how do they get access and benefits from the system, who doesn’t have access to the system, so systemic barriers.” [LS 042].*

Lastly, the subtheme ‘*Organizational barriers’* encapsulates multiple system level barriers faced by HCPs that may hinder their uptake of an asynchronous course on TIC. Leadership staff mentioned that HCPs are already burdened by courses required by their professional and licensing bodies which may prevent them from partaking in additional TIC courses.

*“One is competing priorities, and another is clinician resistance to do online training. I think they would be the two top barriers, and the government needs to promote that this is a good thing.” [LS 38].*

Asynchronous courses imply increased screen time, which can be another barrier for providers already spending hours in front of screens. As such, respondents suggested mandating the training to generate greater uptake.

*“It might be something that we would want to say across the organization we do require this for all providers, and I do think it would be nice in the context of something else.” [LS 038].*

### Curriculum outline

Based on the findings from the study, the research team developed a curriculum outline for a course on universal TIC principles for multidisciplinary healthcare staff (see Additional file 4). The outline consisted of nine major topics, each with specific learning objectives and outcomes. The initial version was shared with interview participants, who provided valuable feedback. Suggestions included incorporating distinctions between the various degrees of trauma, exploring the definition and evolving understanding of trauma and emphasizing risk factors and protective mechanisms in shaping individual responses to trauma. This feedback was used to refine the curriculum outline, ensuring it addressed the nuances and definition of trauma and varied responses to adverse experiences. Feedback from 2 HCPs and 1 leadership staff was incorporated to finalize the curriculum outline on universal TIC skills and strategies.

## Discussion

Our needs assessment study evaluated the preferences, knowledge base, barriers, and facilitators related to a course for multidisciplinary HCPs on trauma-informed care. The findings revealed significant interest for TIC education across diverse HCP groups, consistent with recent research [13, 35]. Patient participants expressed concerns about inadequate trauma-informed care in the healthcare system and a perceived lack of provider knowledge about trauma and its effects. All participant groups consistently emphasized the need for a deeper understanding of how marginalization and cultural factors influence trauma and impact health outcomes. Addressing these issues can foster a paradigm shift within healthcare organizations by tackling societal, cultural, structural, and organizational inequities that might otherwise hinder the delivery of TIC, particularly in diverse and under-resourced settings [9, 55, 77]. TIC education has a particular focus on being a patient-centred, holistic approach that requires a systemic and cultural shift to promote trauma-sensitivity at all levels of care [76].

Interestingly, although many HCP participants reported prior exposure to TIC training and felt confident in their TIC-related knowledge, they still identified a need for additional education. A key finding was the emphasis on knowledge retention and practical application of TIC principles in clinical care, aligning with existing literature that emphasizes the need for effective learning techniques to teach the real-life applicability of TIC skills and strategies [7, 27, 33, 99]. The importance of focusing on rigorous implementation and evaluation methods, as well as understanding the impact of TIC education on patient care outcomes, have been outlined in recent reviews on TIC [6, 61, 75, 80]. The barriers to engaging with TIC education identified by our participants, such as concerns about time, workload, educational burden, accessibility and lacking confidence to address trauma, are consistent with those reported in previous studies [10, 19, 20, 44, 69, 70].

Participants emphasized the importance of universal TIC education for both clinical and non-clinical staff. Despite this, there remains a significant gap in providing comprehensive TIC training across entire healthcare organizations, as most existing TIC educational initiatives are targeted toward specific healthcare providers, rather than including all staff [72, 93]. However, emerging literature indicates a growing trend toward more multidisciplinary training programs [31, 36, 65]. Adopting a multilevel approach that integrates TIC into organizational policies and procedures while engaging the entire workforce is essential for achieving a system-wide transformation [53, 57, 88].

Many participants in our study expressed a strong interest in an asynchronous format for a virtual TIC course. HCPs and leadership staff identified time constraints as a major barrier to TIC education, making an asynchronous delivery model particularly advantageous. This approach addresses time-related concerns and reduces travel costs and scheduling challenges, which are especially pertinent when implementing multidisciplinary educational initiatives [34, 37, 49]. Additionally, an asynchronous format offers opportunities to revisit materials, incorporate multimedia elements to enhance knowledge retention and contribute to the ability to scale an intervention [20, 51]. Our results underscore the importance of developing a broadly applicable course for healthcare providers and show support for an asynchronous learning format. These insights guided the development of a TIC curriculum outline which has received feedback.

After analyzing our findings, we designed a curriculum outline for our TIC course. Our curriculum addresses the intersection of trauma with cultural diversity and systemic inequities, a topic that is crucial to providing TIC and adequate healthcare [47, 48]. This may explain why our interview participants emphasized these aspects, underscoring the need for such content in professional training. Our findings resonate with previously identified educational competencies and needs in TIC. Topics we identified that are similar to those in the literature include understanding the impact and prevalence of trauma and how TIC can mitigate adverse outcomes, acquiring basic self-regulation skills, learning about trauma-informed communication, screening practices and physical examination, recognizing how patient behaviours may be coping mechanisms for trauma and practicing self-care strategies to address secondary traumatic stress [7, 20, 21, 29, 39, 52]. As these studies were conducted in diverse healthcare settings, the similarities in content areas suggest a growing consensus on core topics of TIC education in healthcare.

### Strengths

By employing an explanatory sequential mixed methods design, we gained comprehensive insights from HCPs, patients, and leadership staff. This approach allowed for iterative refinements of our interview guides after analysis of the questionnaire data, which allowed for a more focused exploration of particular data points [26]. HCP interviews, for instance, revealed gaps in the practical application of theoretical knowledge, explaining why many questionnaire respondents expressed low confidence in applying TIC to daily practice despite previous training. We applied Kern’s six-step curriculum development model, incorporating diverse perspectives from the outset to create a curriculum outline that is both practical and aligned with the healthcare community's needs. Kern’s six-step model for curriculum development has demonstrated effectiveness in developing comprehensive courses by keeping the needs of targeted learners at the forefront of curriculum design and addressing barriers highlighted in the needs assessment stage [2, 63, 86]. Another strength of our study was the high questionnaire completion rate and interview data saturation among all stakeholder groups, ensuring robust mixed methods data.

### Limitations

One of the limitations of our study include that participants were recruited from two urban hospitals and did not include healthcare organizations in rural areas or community-based centres, potentially excluding more diverse populations. As well, participation was voluntary, which may have introduced selection bias, particularly among HCPs who already recognize the importance of TIC and are interested in further training. Additionally, the majority of HCP participants self-identified as women, all of which may limit the generalizability of our findings to a more diverse group of healthcare providers. This gender disparity mirrors trends in other TIC studies, which also report higher women-identified participation [10, 55, 84, 96]. The emphasis by participants on the need to ensure that male providers are educated on TIC and improve care for women-identified and gender-diverse patients highlights a significant issue that warrants further exploration.

## Conclusion

Our study underscores a critical gap in TIC knowledge and skills among a broad range of healthcare providers, particularly in the practical application of TIC in everyday clinical practice. By employing a stakeholder-driven design, we identified a strong demand for a TIC training program that not only addressed the specific educational needs of HCPs but also offers a flexible, accessible delivery format that accommodates their schedules. Following Kern’s six-step approach to curriculum development, the next steps will involve completing step 4 by incorporating specific educational strategies aligned with the learning objectives, and step 5, which focuses on implementing the TIC curriculum with the necessary personnel and resources. In future phases, we will fully develop, implement, and evaluate this asynchronous TIC training course to ensure its scalability and practicality to wider hospital staff. This approach will enable the broader adoption of trauma-informed practices, improving care outcomes. Our findings contribute to the growing body of evidence supporting the need for TIC education that emphasizes the translation of theoretical knowledge into actionable clinical practice. Moreover, the study highlights the importance of incorporating key elements such as cultural competence, equitable care, and attention to the unique needs of marginalized communities within TIC education. By focusing on this priority area, our study lays the groundwork for future educational initiatives that are both inclusive and impactful, ensuring that healthcare providers are better equipped to deliver compassionate, trauma-informed care across diverse settings.

## Supplementary Information

Additional file 1. Patient Interview Guide.

Additional file 2. HCP Interview Guide.

Additional file 3. Key Stakeholder Interview Guide.

Additional file 4. TIC Curriculum Outline.

## Data Availability

The datasets used and/or analysed during the current study are available from the corresponding author on reasonable request.

## Abbreviations

TIC: Trauma-informed Care
HCPs: Healthcare Providers
LS: Leadership Staff
Redcap: Research Electronic Data Capture
TTP: Trauma Therapy Program
WCH: Women’s College Hospital
CAMH: The Centre for Addiction and Mental Health
SWs: Social Workers
MDs: Medical Doctors
