# Supplementary material for: A cross-sectional needs assessment for a trauma-informed care curriculum for multidisciplinary healthcare providers
Source: BMC Health Serv Res. 2025 Mar 24;25:426. doi: 10.1186/s12913-025-12568-1 (PMC11931758; doi:10.1186/s12913-025-12568-1)
Supplement: Supplementary file 1 — Additional file 1. Patient Interview Guide. [file 12913_2025_12568_MOESM1_ESM.docx]

Additional File 1: Semi-structured Individual Interview Guide for Patient Participants

**Introduction**
Thank you for agreeing to participate in this individual virtual interview. We are interviewing you to better understand your experiences with trauma-informed care in the healthcare system, and what you would like healthcare providers to learn about trauma and trauma-informed care. We will use the information collected to help design the curriculum for a virtual course on trauma-informed care for multidisciplinary healthcare providers.

There are no right or wrong answers to any of our questions, we are interested in your own thoughts and experiences. We won’t be speaking about the details of your own trauma history today, but rather focusing on your experiences with healthcare providers and healthcare organizations, regarding the delivery of trauma-informed care.

Participation in this study is voluntary and your decision to participate, or not participate, will not have any type of negative effect. The interview should take approximately 45 minutes depending on how much information you would like to share. With your permission, I will audio record the interview because I don’t want to miss any of your comments. All responses will be kept confidential. This means that your de-identified interview responses will only be shared with research team members, and we will ensure that any information we include in our report does not identify you as the respondent. You may decline to answer any question or stop the interview at any time and for any reason. Are there any questions about what I have just explained?

May I turn on the digital recorder?

______________________________________________________________________________

Following the completion of our individual interviews, and analysis of the results from an earlier questionnaire completed by HCPs, we will develop a curriculum map of the proposed TIC curriculum. We would like to send you an email containing the proposed curriculum as an opportunity for you to provide any feedback. Would that be okay?

I will begin by sharing a definition of trauma-informed care provided by the Substance Use and Mental Health Services (SAMSA). Their definition is as follows, “A program, organization, or system that is trauma-informed realizes the widespread impact of trauma and understands potential paths for recovery; recognizes the signs and symptoms of trauma in clients, families, staff, and others involved with the system; and responds by fully integrating knowledge about trauma into policies, procedures, and practices, and seeks to actively resist re-traumatization.”

1. I would like to start by asking some information about you.

What is your age range? (18-24 years old, 25-34 years old, 35-44 years old, 45-54 years old, 55-64 years old, 65 years or older)

What is your gender?

What is your ethnic or racial group?

Any other intersectionality’s or aspects of your identity that you would like to share with us?

1. How familiar are you with the term Trauma-informed Care?
2. Do you feel that you have received what you would consider to be TIC either from individual healthcare providers and/or from healthcare organizations? If yes, what positive aspects stand out to you about the experiences?
3. Have there ever been times when you have felt that the healthcare you were receiving was not being provided in a trauma-informed manner? If yes, what could the healthcare professional or organization have done to improve the experience?
4. We are currently doing an assessment to determine the curriculum for an online course on TIC for multidisciplinary healthcare providers. Are there particular topics or areas about trauma or the delivery of trauma-informed care that you would like to see included in this new curriculum? Interviewer can provide a list of topics if needed:

Knowledge of trauma: definition, prevalence, impact, and recognition.

How to incorporate principles of equity, diversity, and inclusion into trauma-informed care practices, including performing culturally sensitive assessments.

Understanding how common symptoms can be ways of coping with trauma.

Neurobiology of trauma.

Overview of treatment modalities.

How to ask about trauma, respond to patient disclosures, and reduce the potential for retraumatizing when delivering services.

How to implement trauma-informed care in the workplace on a systemic or organizational level.

Trauma-informed skills and strategies for general use in clinical work.

How to correctly use screening and assessment instruments.

1. When you first meet healthcare providers, how often do they tend to ask if you have ever experienced trauma in your lifetime?
2. How would you feel if healthcare providers asked as a standard part of history taking when first meeting you, if you have had experiences in your lifetime that were traumatic that continue to impact you today?
3. Is there anything else that you would like to comment on that I haven’t asked about today or something you would like to expand on from our discussion?

Thank you very much for your time today. We will send you an email in 3-6 months with a copy of the proposed TIC curriculum and would appreciate any comments or feedback that you are able to provide.
