# Supplementary material for: A cross-sectional needs assessment for a trauma-informed care curriculum for multidisciplinary healthcare providers
Source: BMC Health Serv Res. 2025 Mar 24;25:426. doi: 10.1186/s12913-025-12568-1 (PMC11931758; doi:10.1186/s12913-025-12568-1)
Supplement: Supplementary file 2 — Additional file 2. HCP Interview Guide. [file 12913_2025_12568_MOESM2_ESM.docx]

Additional File 2: Semi-Structured Interview Guide for HealthCare Providers

**Introduction**
Thank you for agreeing to participate in this individual interview. We are interviewing you to better understand what you think about a virtual, TIC course for multidisciplinary healthcare providers that will be asynchronous (i.e., is completed independently). We would like to understand how we can improve the way we provide education on the topic of TIC principles and practices. So, there are no right or wrong answers to any of our questions, we are interested in your own thoughts and experiences.

Participation in this study is voluntary. The interview should take approximately 45 minutes depending on how much information you would like to share. With your permission, I would like to audio record the interview because I don’t want to miss any of your comments. All responses will be kept confidential. This means that your de-identified interview responses will only be shared with research team members, and we will ensure that any information we include in reports or publications do not identify you as the respondent. You may decline to answer any question or stop the interview at any time and for any reason. Are there any questions about what I have explained so far?

May I turn on the digital recorder?

______________________________________________________________________________

Following the completion of our individual interviews, and analysis of the results from an earlier questionnaire completed by HCPs, we will develop a curriculum map of the proposed TIC curriculum. We would like to send you an email containing the proposed curriculum as an opportunity for you to provide any feedback. Would that be okay?

I will begin by sharing a definition of trauma-informed care provided by the Substance Use and Mental Health Services (SAMSA). Their definition is as follows, “A program, organization, or system that is trauma-informed realizes the widespread impact of trauma and understands potential paths for recovery; recognizes the signs and symptoms of trauma in clients, families, staff, and others involved with the system; and responds by fully integrating knowledge about trauma into policies, procedures, and practices, and seeks to actively resist re-traumatization.”

1. I would like to start by asking some information about you.

What is your age range? (18-24 years old, 25-34 years old, 35-44 years old, 45-54 years old, 55-64 years old, 65 years or older)

What is your gender?

What is your ethnic or racial group?

Any other intersectionalities or aspects of your identity that you would like to share with us?

1. How long have you been working in the healthcare field?
2. What is your current field of occupation? How long have you been employed in this position?
3. How familiar are you with the concept of TIC?
4. What, if any, educational experiences or training have you already received around TIC? This could include when you were a student, through CFD, or external trainings. What did you find the most helpful or memorable about these educational experiences?
5. Are you aware of any current resources or educational opportunities to learn more about TIC at your workplace?
6. Where are the areas that you feel least confident or worried about when it comes to working with patients who have a history of trauma?
7. Have you learned TIC skills or strategies that you can apply with your patient population? If so, what? If not, what do you imagine might be helpful?
8. Do you have any concerns about the concept of TIC in general, or around receiving additional education about TIC?
9. How comfortable are you with asynchronous virtual courses? What have been the strengths and drawbacks in your experience?
10. If you were to take a virtual course, what type of multimedia might you prefer? Some examples include podcasts, videos, animations, simulations, handouts, infographics, etc.
11. How much time would you be willing to dedicate to completing a virtual TIC course?
12. What barriers exist that would make it challenging to complete a virtual, asynchronous course on TIC?
13. Is there anything else that you would like to comment on that I haven’t asked about today?

Thank you very much for your time today. We will send you an email in 3-6 months with a copy of the proposed TIC curriculum and would appreciate any comments or feedback that you are able to provide.
