# Supplementary material for: A cross-sectional needs assessment for a trauma-informed care curriculum for multidisciplinary healthcare providers
Source: BMC Health Serv Res. 2025 Mar 24;25:426. doi: 10.1186/s12913-025-12568-1 (PMC11931758; doi:10.1186/s12913-025-12568-1)
Supplement: Supplementary file 3 — Additional file 3. Key Stakeholder Interview Guide. [file 12913_2025_12568_MOESM3_ESM.docx]

Additional File 3: Semi-structured Individual Interview Guide for Key Stakeholders

**Introduction**
Thank you for agreeing to participate in this individual interview. We are interviewing you to better understand what you, as a key stakeholder, think about a virtual course on trauma-informed care (TIC) for multidisciplinary healthcare professional. The course will likely be asynchronous which means it is completed independently and is self-paced. We would like to understand how we can improve the way we provide education to healthcare providers on the topic of TIC principles and practices. So, there are no right or wrong answers to any of our questions, we are interested in your own thoughts and experiences.

Participation in this study is voluntary. I will ask you 12 questions, and the interview should take approximately 30-45 minutes depending on how much information you would like to share. With your permission, I would like to audio record the interview because I don’t want to miss any of your comments. All responses will be kept confidential. This means that your de-identified interview responses will only be shared with research team members, and we will ensure that any information we include in reports or publications do not identify you as the respondent. You may decline to answer any question or stop the interview at any time and for any reason. Are there any questions about what I have explained so far?

May I turn on the digital recorder?

_______________________________________________________________________

1. I’d like to start by asking some demographic questions.

What is your gender?

What is your ethnic or racial group?

What is your highest level of education?

What is your age group? 18-30, 31-45, 46-59, 60 +

Are there any other intersectionality’s or aspects of your identity that you would like to share with us?

1. I understand your role is (x). How long have you been working in the healthcare field, and how long have you been in this current role?
2. In terms of your familiarity with the concept of Trauma-informed Care would you say that you are not at all familiar, a little familiar, unsure, somewhat familiar, very familiar?

I will now ask you some questions specific to Trauma-informed Care. I will begin by sharing a definition of trauma-informed care provided by the Substance Use and Mental Health Services (SAMSA). Their definition is as follows, “A program, organization, or system that is trauma-informed realizes the widespread impact of trauma and understands potential paths for recovery; recognizes the signs and symptoms of trauma in clients, families, staff, and others involved with the system; and responds by fully integrating knowledge about trauma into policies, procedures, and practices, and seeks to actively resist re-traumatization.”

1. What, if any, educational experiences or training have you already received around TIC? This could include when you were a student, through continuing faculty development, or external trainings. What did you find the most helpful or memorable about these educational experiences?
2. Do you have any concerns about the concept of TIC in general?
3. Are you aware of any current resources or educational opportunities for HCPs or non-HCP staff to learn more about TIC at your organization?
4. We plan on making this asynchronous virtual TIC course available to our learners in mental health. If the course was made more widely available to staff at WCH, what do you think would be the ideal length of time to complete the course from start to finish?
5. In this phase of the study, we are focusing on developing a TIC virtual course for HCP’s. What are your thoughts on the idea of offering this course more broadly to the WCH community, for example to administrative staff and non-HCP employees?
6. What do you think the impact of a TIC curriculum for multidisciplinary healthcare providers could be for patient care outcomes and/or HCP wellness?
7. What are the barriers to the implementation of the virtual TIC course at WCH/CAMH or in your area of practice?
8. Are there any particular topics that you would like to see covered in this TIC course?
9. Is there anything else that you would like to comment on that I haven’t asked about today?

Thank you very much for your time today. Our aim is to complete a thematic analysis of our responses from individual interviews with HCPs, patients, and key stakeholders. Then we will develop a curriculum map of the proposed TIC curriculum.

We would like to email you in ~3 months with a draft of an outline for our TIC curriculum and would appreciate any comments or feedback that you are able to provide. Providing feedback would be optional. Would that be okay? Yes/No.
