# Supplementary material for: A cross-sectional needs assessment for a trauma-informed care curriculum for multidisciplinary healthcare providers
Source: BMC Health Serv Res. 2025 Mar 24;25:426. doi: 10.1186/s12913-025-12568-1 (PMC11931758; doi:10.1186/s12913-025-12568-1)
Supplement: Supplementary file 4 — Additional file 4. TIC Curriculum Outline. [file 12913_2025_12568_MOESM4_ESM.docx]

**Additional File 4: TIC Curriculum Outline**

| **Topics** | **Learning Outcome** | **Learning Objectives** |
| --- | --- | --- |
| Topic 1: Introduction to Trauma-informed Care | - Knowledge: understand the importance of TIC in relation to health outcomes and organizational well-being.  - Competence/Skill: Correctly identify the 6 principles of TIC.  - Knowledge: Understand how re- traumatization in healthcare can negatively impact health and how to minimize this risk. | -Define the concept of trauma and trauma-informed care.  -Name 6 key principles of trauma- informed care.  -Describe the term re-traumatization. |
| Topic 2: Introduction to Trauma | - Understanding Adverse Childhood Experiences (ACEs) and key findings related to that study.  - Understand the implications of trauma prevalence in Canada.  - Learn about the importance and relevance of learning about TIC in healthcare environments. | - Describe different types of trauma experiences.  - Recall the prevalence of trauma in Canada.  - Name at least 5 ways in which trauma can impact physical health and 5 ways in which trauma can impact mental health. |
| Topic 3: Trauma Symptoms & Comorbidity | - Recognize common mental and physical health symptoms, behaviors, and comorbid (often associated) diagnosis’ that can be related to trauma.  - To understand that symptoms and behaviors can often be coping strategies that individuals who have experienced trauma may use. | - List 10 symptoms that can be related to trauma experiences.  - Name 5 mental health diagnoses commonly associated with trauma.  - List 10 common behaviors that can be a coping mechanism to manage trauma-related symptoms. |
| Topic 4: Neurobiology | - Identify and differentiate between survival responses in the context of trauma.  - Understand and describe impact of trauma on cognition (e.g. thought process, and perception).  - How trauma relates to altered neurobiological pathways and its impact on person’s behavior. | - Practice explaining the role of the amygdala in trauma.  - Identify examples of the survival responses (fight, flight, freeze, collapse/submit).  - Describe the impact that trauma can have on cognition. |
| Topic 5: When/How to ask about Trauma | - Learn communication skills when asking about trauma and/or responding to patient/client/colleague disclosures to create a supportive, non-retraumatizing environment.  - To provide a basic understanding of, and ability to differentiate between, various tools used to assess trauma. | - Practice how to ask about trauma and respond to patient/client/colleague disclosures.  - Name 3 examples of universal screening questions for trauma.  - List 3 tools to assess trauma. |
| Topic 6: Physical touch and TIC | - To provide a basic understanding of the definition and importance of consent.  - Learn how to apply the principles of consent in healthcare practices, including during physical exams. | - List 3 important components of consent.  - Describe 3 methods to ask for consent around physical touch in a trauma- informed manner.  - Summarize 5 tips for how to perform physical exams utilizing a trauma- informed approach. |
| Topic 7: Emotions and TIC | - Understanding and utilizing the basic skills and strategies to help with emotion regulation.  - Learn how to apply trauma-informed non- verbal communication skills.  - Understand trauma-informed conflict resolution skills and strategies. | - Practice 5 grounding tools to manage emotional dysregulation.  - Analyze the use of 5 non-verbal communication skills in video demonstrations.  - Learn 3 trauma-informed conflict resolution skills. |
| Topic 8: Equity, Diversity, Inclusion & TIC | - Demonstrate the benefits that can result when a healthcare organization uses of inclusive TIC approaches.  - Equip healthcare staff with strategies to engage with the patient population, to prevent re-traumatization and promote a healing atmosphere. | - Name 5 practical approaches to create a welcoming and inclusive space.  - Describe 5 common societal, cultural/organizational barriers that can impact the provision of trauma- informed care. |
| Topic 9: TIC in the Workplace | - Understanding of the emotional and psychological impact of vicarious trauma.  - Demonstrate self-care strategies and identify skills for minimizing and avoiding burnout.  - Highlight the importance of having a trauma-informed workplace and provide effective organizational tools and strategies to do so. | - Define the term vicarious trauma. - Explain the concept of burnout.  - Learn 8 strategies for healthcare staff self-care.  - List 5 approaches that organizations can implement to align with trauma- informed practices. |
